# Pharmacokinetics and Pharmacodynamics of Fosfomycin and Its Activity against Extended-Spectrum-β-Lactamase-, Plasmid-Mediated AmpC-, and Carbapenemase-Producing Escherichia coli in a Murine Urinary Tract Infection Model

**DOI:** 10.1128/AAC.02560-17

**Published:** 2018-05-25

**Authors:** Ilya Nikolaevich Zykov, Ørjan Samuelsen, Lotte Jakobsen, Lars Småbrekke, Dan I. Andersson, Arnfinn Sundsfjord, Niels Frimodt-Møller

**Affiliations:** a Norwegian National Advisory Unit on Detection of Antimicrobial Resistance, Department of Microbiology and Infection Control, University Hospital of North Norway, Tromsø, Norway; b Research Group for Host-Microbe Interactions, Department of Medical Microbiology, Faculty of Health Sciences, UiT—The Arctic University of Norway, Tromsø, Norway; c Microbial Pharmacology and Population Biology Research Group, Department of Pharmacy, Faculty of Health Sciences, UiT—The Arctic University of Norway, Tromsø, Norway; d Department of Microbiology and Infection Control, Statens Serum Institut, Copenhagen, Denmark; e Clinical Pharmacy and Pharmacoepidemiology (IPSUM), Department of Pharmacy, Faculty of Health Sciences, UiT—The Arctic University of Norway, Tromsø, Norway; f Department of Medical Biochemistry and Microbiology, Uppsala University, Uppsala, Sweden; g Department of Clinical Microbiology, Copenhagen University Hospital, Rigshospitalet, Copenhagen, Denmark

**Keywords:** reviving old drugs, CTX-M, VIM, NDM, multidrug resistant, *in vivo*, time-kill, PK/PD, UTI, UTI model, fosfomycin, *in vivo* model

## Abstract

Fosfomycin has become an attractive treatment alternative for urinary tract infections (UTIs) due to increasing multidrug resistance (MDR) in Escherichia coli. In this study, we evaluated the pharmacokinetic (PK) and pharmacodynamic (PD) indices of fosfomycin and its *in vivo* activity in an experimental murine model of ascending UTI. Subcutaneous administration of fosfomycin showed that the mean peak plasma concentrations of fosfomycin were 36, 280, and 750 mg/liter following administration of a single dose of 0.75, 7.5, and 30 mg/mouse, respectively, with an elimination half-life of 28 min, and urine peak concentrations of 1,100, 33,400, and 70,000 mg/liter expected to be sustained above 1 mg/liter (MIC of the test strain, NU14) for 5, 8, and 9.5 h, respectively. The optimal PK/PD indices for reducing urine colony counts (number of CFU per milliliter) were determined to be the area under the concentration-time curve/MIC from 0 to 72 h and the maximum concentration/MIC on the basis of the dose-dependent bloodstream PK and the results of an evaluation of six dosing regimens. With a dosing regimen of 15 mg/mouse twice (every 36 h), fosfomycin significantly reduced the number of CFU per milliliter of all susceptible strains in urine, including clinical MDR strains, except for one clinical strain (*P* = 0.062). Variable degrees of reduction were observed in the bladder and kidneys. No significant reductions in the number of CFU per milliliter were observed with the resistant strains. In conclusion, fosfomycin shows concentration-dependent *in vivo* activity, and the results suggest that fosfomycin is an effective alternative to carbapenems in treating MDR E. coli in uncomplicated UTIs. The data on the effectiveness of fosfomycin against the MDR isolates along with the results of PK/PD modeling should facilitate the further development of improved recommendations for its clinical use.

## INTRODUCTION

Urinary tract infections (UTIs) may progress to bloodstream infections (1), and they account for ∼40% of hospital-acquired cases of sepsis (2, 3). Escherichia coli is responsible for 75 to 90% cases of community-acquired UTIs (4–7). The increase in antimicrobial resistance and multidrug resistance (MDR) among E. coli isolates (i.e., extended-spectrum-β-lactamase [ESBL]-producing E. coli isolates) is currently limiting treatment options for UTIs (8). This could lead to the more extensive use of carbapenems, which are reserved for use against other complicated infections (9). In addition, carbapenemase-producing organisms are now spreading worldwide (10, 11). The lack of effective antimicrobials due to the emergence of antimicrobial resistance inflates the use of last-resort antimicrobials for common infections, such as UTIs. Alternative therapeutic options for UTIs are therefore urgently needed (12).

As there are a very limited number of new antimicrobials in the pipeline, it has been suggested that an alternative approach is to reevaluate the efficacy of old antimicrobials to extend the set of drugs available for the treatment of MDR infections (13–16). One such agent is fosfomycin, a broad-spectrum bactericidal agent that has been suggested to be an alternative treatment option for infections caused by MDR Gram-negative bacteria (17). Fosfomycin acts on the cell wall by inactivating enolpyruvate transferase, thereby blocking the condensation of UDP-*N*-acetylglucosamine with *p*-enolpyruvate (18).

Recent studies have shown that fosfomycin exhibits potent *in vitro* activity against both non-MDR and MDR Enterobacteriaceae, including ESBL- and carbapenemase-producing isolates (18–20). However, increasing frequencies of fosfomycin resistance have been observed in some countries where fosfomycin is used (18, 20). The emergence of resistance during fosfomycin monotherapy occurs rapidly *in vitro* but is rarely observed *in vivo* (21). Data on the pharmacokinetic (PK) and the pharmacodynamic (PD) behavior of fosfomycin are somewhat conflicting among existing studies (22–26). Docobo-Perez et al. (22) suggest insufficient evidence on efficacy to be among the factors discouraging the use of fosfomycin as a treatment option. Therefore, reevaluation of the *in vivo* activity and PK/PD properties of fosfomycin is required to develop an effective dosing regimen that complies with current standards and that is applicable to the current bacterial population (15, 16, 21). To our knowledge, no *in vivo* studies have investigated the PK/PD of fosfomycin in UTIs during the past 2 decades.

Thus, the objectives of this study were to elucidate the predictive PK/PD index for fosfomycin in an experimental model of ascending UTI, identify the dose that targets appropriate exposure toward E. coli strains with decreased susceptibility to fosfomycin, and investigate the *in vivo* activity of fosfomycin against MDR ESBL-, plasmid-mediated AmpC-, and/or carbapenemase-producing E. coli *in vivo*.

## RESULTS

### Bacterial strain characteristics.

The characteristics of the strains used in this study are summarized in Table 1. On the basis of the results of whole-genome sequencing (WGS) analysis, no resistance determinants were identified in the NU14 strain. The sequence type (ST) of NU14 was determined to be ST1231. The MICs of fosfomycin for NU14 and NU14-derived strains DA6313, DA6328, and DA6401 were determined to be 1, 2, 128, and >1,024 mg/liter, respectively.

**TABLE 1:** Characteristics of the E. coli strains used in the study

Strain	Specimen or origin	Fosfomycin MIC (mg/liter)	Resistance gene profile	MLST type
NU14	Urine	1		ST1231
DA6313	NU14 derivative	2	*ptsI* deletion	ST1231
DA6328	NU14 derivative	128	*glpT* missense mutation	ST1231
DA6401	NU14 derivative	>1,024	*uhpT* missense mutation	ST1231
K4-40	Wound	2	*aadA1*, *aac(6′)Ib-cr*, *bla*_CTX-M-15_, *bla*_OXA-1_, *bla*_TEM-1B_, *catB3*, *dfrA1*, *erm*(B), *mph*(A), *strA*, *strB*, *sul2*, *tet*(A)	ST167
K5-08	Urine	0.5	*aadA5*, *bla*_CTX-M-14_, *dfrA17*, *sul2*, *tet*(A)	ST2016
K26-07	Urine	2	*bla*_CMY-2_	ST420
K71-77	Blood culture	2	*aac(3)-IId*, *aac(6′)Ib-cr*, *aacA4*, *bla*_CMY-6_, *bla*_NDM-1_, *bla*_OXA-1_, *catB3*, *rmtC*, *sul1*	ST410
50639799	Urine	0.5	*aac(3)-IIa*, *aac(6′)Ib-cr*, *aadA24*, *bla*_CMY-4_, *bla*_CTX-M-15_, *bla*_OXA-1_, *bla*_VIM-29_, *catB3*, *dfrA1*, *floR*, *strA*, *strB*, *sul2*, *tet*(A)	ST6355
P14-63	Urine	512	*bla*_CTX-M-3_, *bla*_TEM-1B_, *fosA*	ST131

Five of the clinical MDR E. coli isolates selected for the *in vivo* activity studies were susceptible to fosfomycin with MICs of 0.5 to 2 mg/liter, while isolate P14-63 was resistant with an MIC of 512 mg/liter. Two of the clinical isolates were carbapenemase producers harboring *bla*_NDM-1_ (strain K71-77) or *bla*_VIM-29_ (strain 50639799). Three isolates were ESBL producers harboring *bla*_CTX-M-15_, *bla*_CTX-M-14_, or *bla*_CTX-M-3_, while the isolate with a plasmid-mediated AmpC harbored *bla*_CMY-2_. The fosfomycin-resistant P14-63 isolate harbored the *fosA* gene. Multilocus sequence typing (MLST) analysis showed that the isolates were diverse with respect to sequence types, with the sequence types of the isolates including ST167, ST2016, ST420, ST410, ST6355, and ST131.

### *In vitro* time-kill studies.

**FIG 1:**
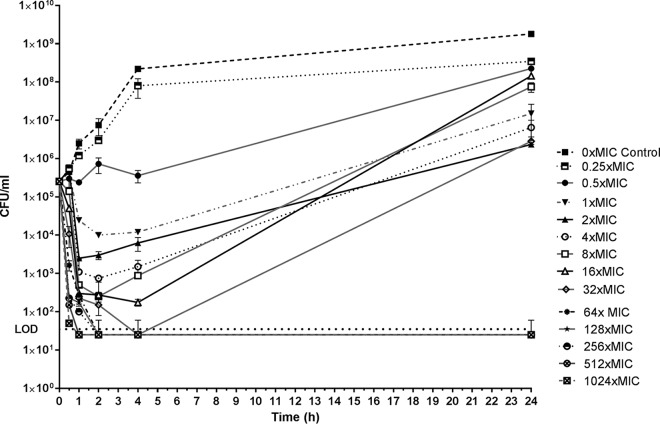
*In vitro* time-kill curves with fosfomycin against fosfomycin-susceptible E. coli NU14 (MIC, 1 mg/liter). The graph shows the viable counts as the log_10_ number of CFU per milliliter at time points of 0 h, 30 min, 1 h, 2 h, 4 h, and 24 h. The horizontal dotted line shows the limit of detection (LOD).

**FIG 2:**
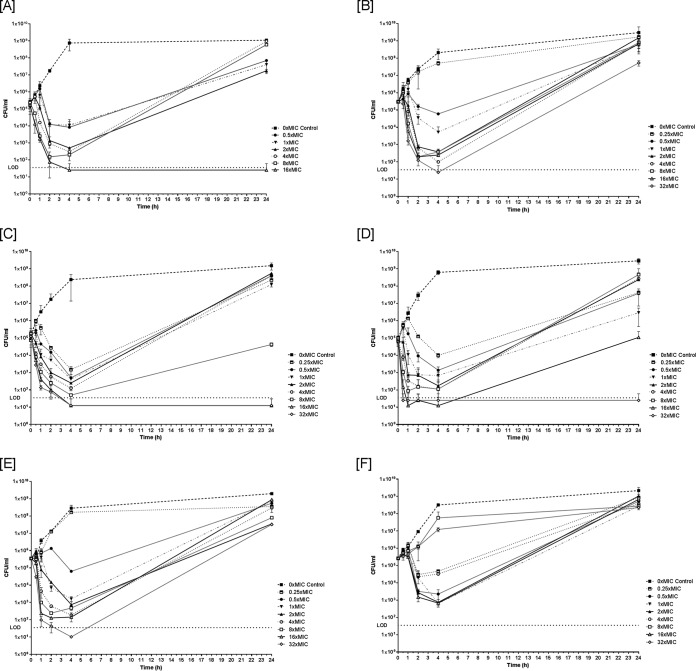
*In vitro* time-kill curves with fosfomycin against E. coli clinical isolates K4-40 (MIC, 2 mg/liter) (A), K5-08 (MIC, 0.5 mg/liter) (B), K26-07 (MIC, 2 mg/liter) (C), K71-77 (MIC, 2 mg/liter) (D), 50639799 (MIC, 0.5 mg/liter) (E), and P14-63 (MIC, 512 mg/liter) (F). The graphs show viable counts as the log_10_ number of CFU per milliliter at time points of 0 h, 30 min, 1 h, 2 h, 4 h, and 24 h. The horizontal dotted lines show the limit of detection (LOD).

*In vitro* time-kill studies with NU14 (Fig. 1) and the susceptible clinical strains (Fig. 2A to E) at concentrations of 1× to 8× MIC showed an initial rapid bactericidal effect up to 2 h, followed by regrowth at 24 h. At concentrations of 16× to 32× MIC (64× MIC and higher for NU14), bacterial counts reached levels below the limit of detection (LOD; ≥50 CFU/ml) at 2 to 4 h. No regrowth was observed at 24 h for any of the susceptible strains, except for K5-08 and 50639799. For NU14, MIC testing of the subsequently isolated colonies (at time points of 0 h, 4 h, and 24 h) showed 8- to 32-fold increases in the MIC at time points of 4 h and 24 h, whereas there was no increase in the MIC for colonies from the control tube. The MICs for the isolated colonies with an increased MIC remained stable after five passages on nonselective Mueller-Hinton (MH) agar (data not shown).

For the resistant clinical strain P14-63 (Fig. 2F), transient killing was soon followed by regrowth. After 24 h, regrowth was observed irrespective of the fosfomycin concentration. For this strain, no dependence between the fosfomycin concentration and the rate of killing was found (i.e., lower concentrations could result in killing rates initially higher than those achieved with higher concentrations of fosfomycin; in the case of 8× MIC and 32× MIC, the growth rates were close to the rates observed for the control). The maximal bactericidal effect did not exceed >2 log_10_ CFU/ml of the initial number of CFU per milliliter.

### PK/PD of fosfomycin. (i) Pharmacokinetics.

**FIG 3:**
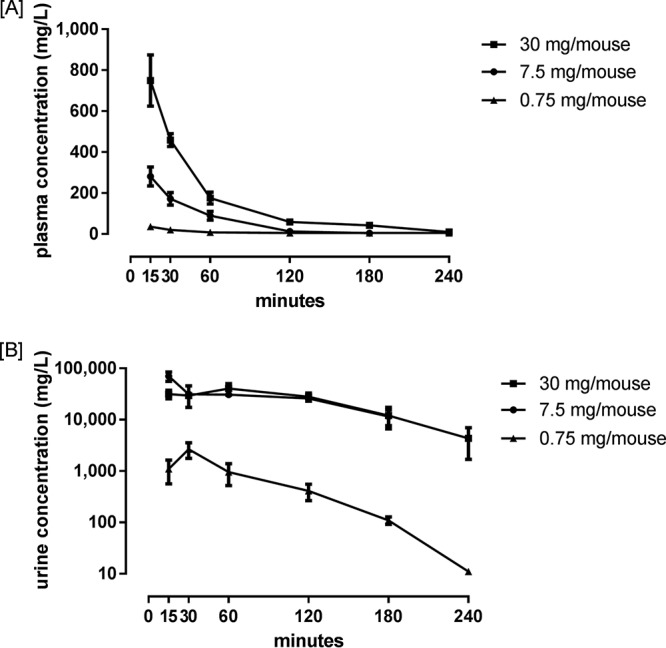
Fosfomycin concentrations (in milligrams per liter) in plasma (A) and urine (B) in OF-1 mice following subcutaneous administration of single doses of 30, 7.5, and 0.75 mg/mouse. The data are presented as the mean for three mice at each time point. Error bars represent SDs.

The plasma and urine concentrations of fosfomycin were measured in mice after single subcutaneous (s.c.) doses of 0.75, 7.5, and 30 mg/mouse. Peak fosfomycin plasma concentrations were 36, 280, and 750 mg/liter for the respective doses (Fig. 3A). The mean elimination half-life was 28 min. In urine, peak fosfomycin concentrations of 33,400 and 70,000 mg/liter were reached after 15 min with the 7.5- and 30-mg/liter doses, respectively (Fig. 3B). After 15 min, the measured concentrations of fosfomycin for the 7.5- and 30-mg/liter doses were similar, and the two doses followed the same elimination pattern. For the 0.75-mg dose, a peak urine concentration of 1,100 mg/liter was reached after 30 min.

### (ii) Dose fractionation and calculation of PK/PD indices.

On the basis of the results of the PK analysis, six dose regimens were designed (Table 2), in order to produce variations in bloodstream PK/PD indices: 30 and 7.5 mg/mouse in a single dose, 15 mg/mouse twice (every 36 h [q36h]), 1.88 and 0.47 mg/mouse every 6 h, and 0.47 mg/mouse every 12 h (q12h). Treatment was initiated at 24 h postinfection, and the treatment period was 72 h for all doses used. PK/PD indices for the NU14 strain were calculated using the systemic drug concentrations. The cumulative percentage of a 72-h period that the drug concentration exceeded the MIC (percent *T*_>MIC_) ranged from 4 to 42%, the area under the concentration-time curve (AUC)/MIC ratios ranged from 607 to 79 h^−1^, and the maximum concentration (*C*_max_)/MIC (for doses of 30 mg and 7.5 mg, the actual measured values were used to calculate *C*_max_/MIC) ratios ranged from 750 to 22 (Table 2).

**TABLE 2:** Fosfomycin dosing regimens, based on bloodstream PK data, applied in the PK/PD study in the experimental UTI model^*a*^

Dose (mg/mouse)	Dosing interval (h)	No. of doses per 72-h treatment interval	Total dose (mg)	Value of the following PK/PD index:		
				*T*_>MIC_ (%)	AUC/MIC (h^−1^)	*C*_max_/MIC
30	72	1	30	9	607	750
15	36	2	30	14	727	468
7.5	72	1	7.5	4	212	281
1.88	6	12	22.56	42	635	78
0.47	6	12	5.64	30	158	22
0.47	12	6	2.82	15	79	22

a Treatment was initiated at 24 h postinfection, and the treatment period was 72 h.

### (iii) PK/PD analysis.

**FIG 4:**
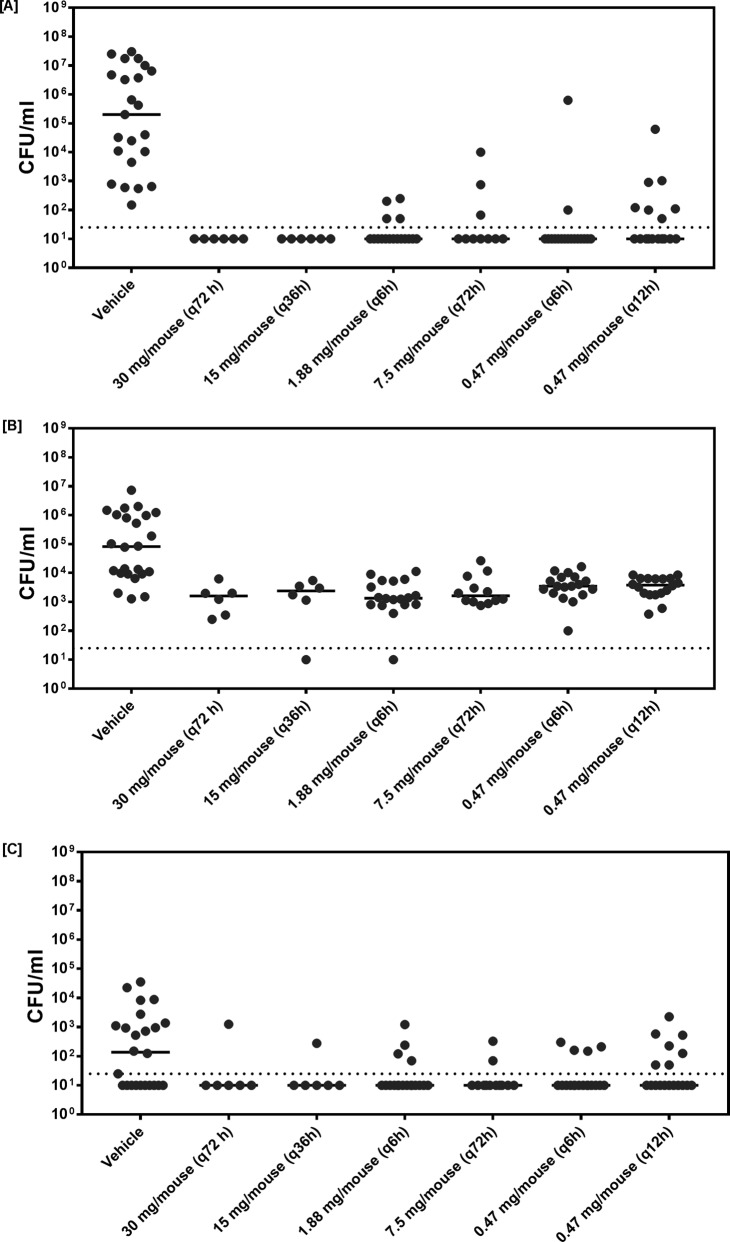
Study of the effect of treatment with six fosfomycin dosing regimens (milligrams per mouse) against E. coli NU14 (MIC, 1 mg/liter). The bacterial counts (number of CFU per milliliter) from urine (A), homogenized urine bladder (B), and homogenized kidneys (C) in mice with UTIs at day 5 after the inoculation are shown. Each point indicates the number of CFU per milliliter in a single animal. Solid horizontal lines represent the median bacterial count for each group, and horizontal dotted lines represent the LOD.

The *in vivo* activity of the six dose regimens against the NU14 strain was further investigated to estimate the predictive value of the PK/PD indices for a bactericidal effect. For all doses tested, the median number of CFU per milliliter in urine and kidneys was reduced to below the LOD (50 CFU/ml) (Fig. 4A and C). However, for some mice and on the basis of the dose, counts (number of CFU per milliliter) above the LOD were observed in the urine of a fraction of the mice in each separate experiment, with the fractions ranging from 0% (for a single dose of 30 mg/mouse and two doses of 15 mg/mouse [q36h]) to 55.6% (for a dose of 0.47 mg/mouse twice a day [q12h]) of the mice (Fig. 4A). This was also observed for the counts (number of CFU per milliliter) in the kidneys, in which the fraction of mice with counts (number of CFU per milliliter) above the LOD ranged from 16.6% (for doses of 7.5 and 30 mg/mouse) to 38.8% (for a dose of 0.47 mg/mouse q12h) (Fig. 4C). In the bladders, none of the median counts (number of CFU per milliliter) fell below the LOD, but a reduction in median counts (number of CFU per milliliter) of up to ∼2 log_10_ compared to the counts for the control was observed for all doses tested (Fig. 4B).

**FIG 5:**
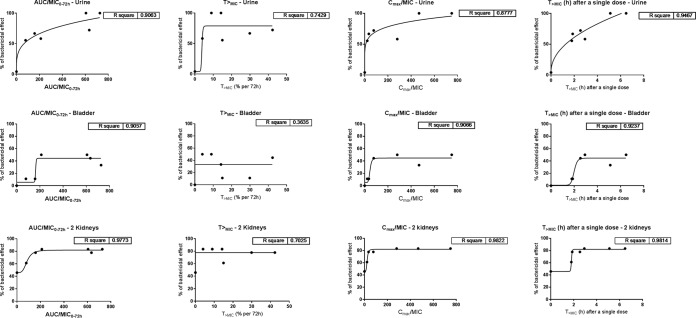
Relationship between AUC/MIC_0–72_, percent *T*_>MIC_ (percent per 72 h), *C*_max_/MIC, and *T*_MIC_ (in hours) after a single dose, based on plasma drug concentrations (protein binding is assumed to be 0%) and fosfomycin efficacy against E. coli NU14 in the experimental UTI model. The dosing regimens applied for the efficacy study are listed in Table 2. Six to 18 mice were used for the investigation of each dose. *R*^2^ represents the goodness of fit, as calculated in GraphPad Prism software.

For all infection sites, the PD indices with the best correlation with *in vivo* activity were the AUC/MIC from 0 to 72 h (AUC/MIC_0–72_) and *C*_max_/MIC (Fig. 5). The percent *T*_>MIC_ for 72 h had minimal, if any, influence on the *in vivo* activity (*R*^2^ = 0.74, 0.36, and 0.7 for urine, bladder, and kidneys, respectively). However, the amount of time that the concentration exceeded the MIC (*T*_>MIC_ in hours) for the first injection (which could also serve as a surrogate for *C*_max_/MIC) also correlated well with the bactericidal effect. The optimal AUC/MIC_0–72_ ratio appeared to be >600 h^−1^ for urine (*R*^2^ = 0.91) and >200 h^−1^ for the bladder and kidneys (*R*^2^ = 0.91 and 0.97, respectively). The optimal values of *C*_max_/MIC were >450 for urine (*R*^2^ = 0.88) and >280 for kidneys and bladder (*R*^2^ = 0.91 and 0.98, respectively). On the basis of this finding, the treatment associated with the best *in vivo* activity (15 mg of fosfomycin per mouse twice [q36h]) was selected for further studies with clinical strains. This dose was calculated on the basis of a surface area of a mouse to be 70 cm^2^, which correlates with a surface area of 17,200 cm^2^ in a 70-kg human. The calculation results in a human dose of 3.6 g, which is close to the standard dose of 3 g fosfomycin used for treating UTIs in most clinical studies (27, 28).

### *In vivo* activity studies.

Both the NU14-derived isogenic and clinical MDR strains were confirmed to be type 1 fimbria positive and virulent in the murine UTI model (data not shown).

### (i) NU14 and isogenic NU14-derived strains.

**FIG 6:**
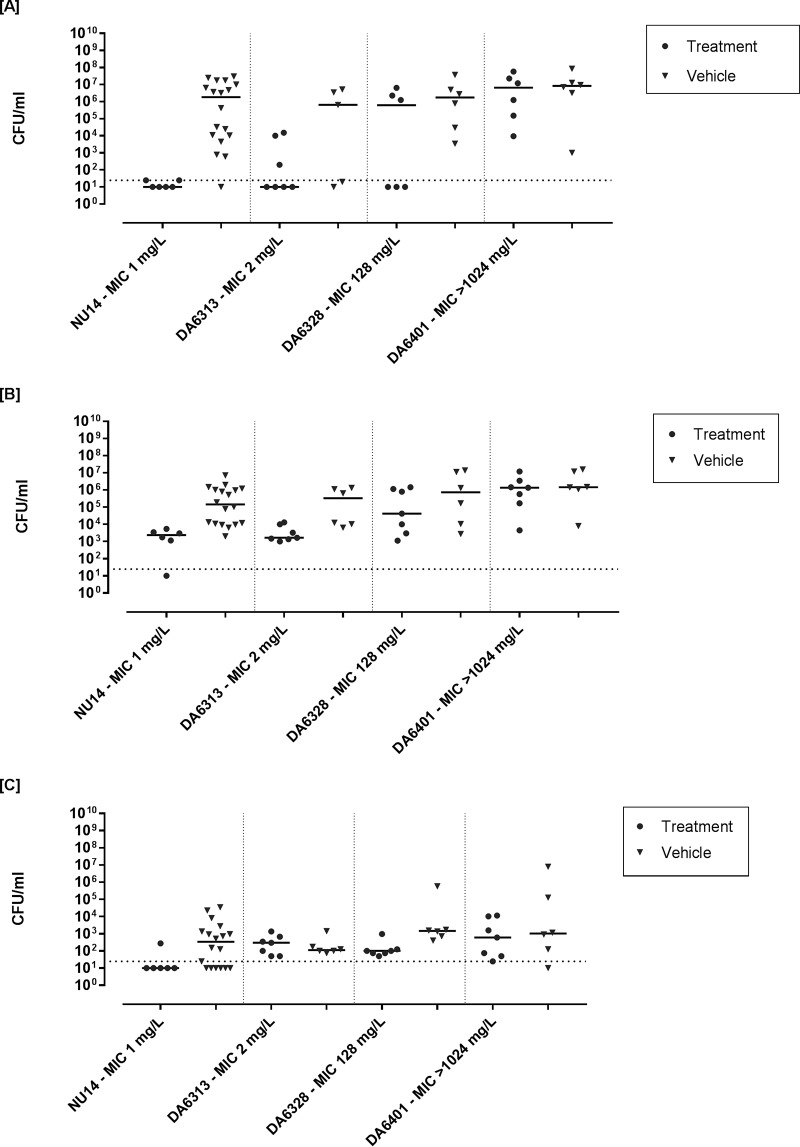
Bacterial counts (number of CFU per milliliter) from urine (A), homogenized urine bladder (B), and homogenized kidneys (C) of OF-1 mice treated for 3 days at 15 mg/mouse twice (q36h) or saline (control) after infection with isogenic E. coli strains with decreasing susceptibility to fosfomycin. Solid horizontal lines represent the median bacterial count for each group, and horizontal dotted lines represent the LOD.

Using the dose of 15 mg/mouse twice (q36h), a reduction in the median number of CFU per milliliter was observed only with fosfomycin-susceptible strain NU14 in urine (5.3-log reduction, *P* < 0.0001), bladder (4.9-log reduction, *P* < 0.0006), and kidneys (2.13-log reduction, *P* = 0.063) and with fosfomycin-susceptible strain DA6313 in urine (5.8-log reduction, *P* = 0.1326) (Fig. 6A) and bladder (1.7-log reduction, *P* = 0.014) (Fig. 6B). No reduction was observed in kidneys (Fig. 6C). For fosfomycin-resistant strains DA6328 and DA6401, no significant reduction in the median counts (number of CFU per milliliter) was observed at any infection site, except that a significant reduction in the number of CFU per milliliter was observed in the kidneys for DA6328 (1.16 log reduction, *P* = 0.041) (Fig. 6C).

### (ii) Clinical MDR strains.

**FIG 7:**
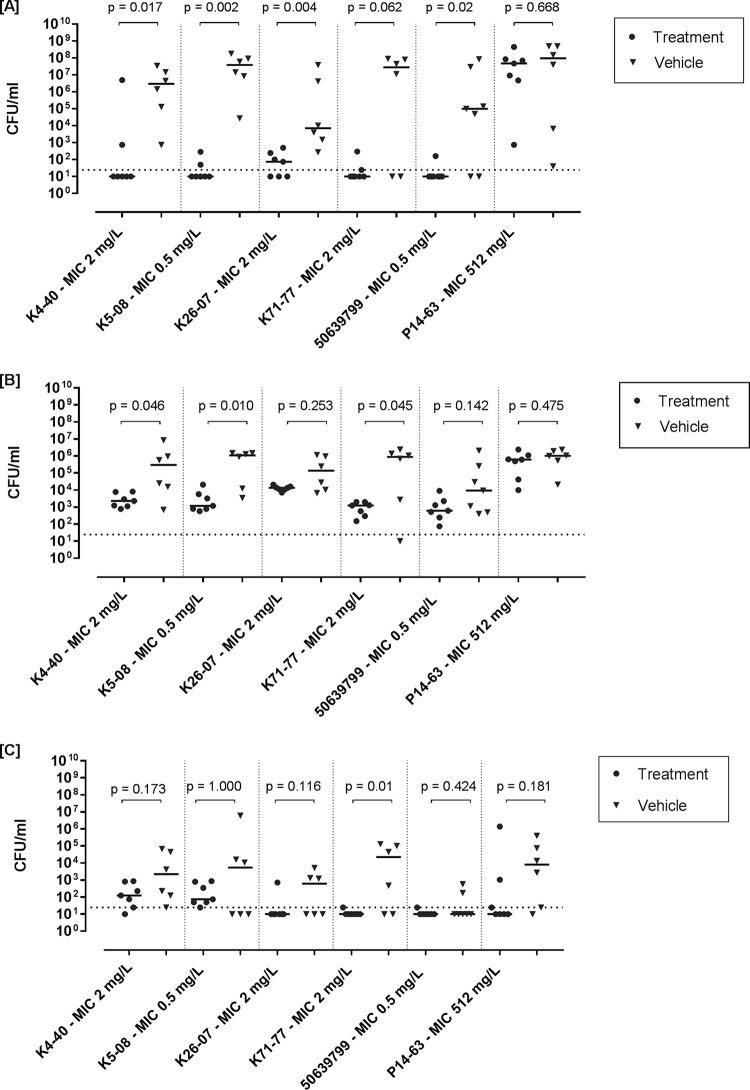
Bacterial counts (number of CFU per milliliter) from urine (A), homogenized urine bladder (B), and homogenized kidneys (C) of OF-1 mice treated for 3 days at 15 mg/mouse twice (q36h) or saline (control) after infection with virulent clinical E. coli isolates with various degrees of fosfomycin susceptibility. Solid horizontal lines represent the median bacterial count for each group, and horizontal dotted lines represent the LOD.

The applied treatment regimen significantly reduced the counts (number of CFU per milliliter) in urine compared to those achieved with the vehicle for all fosfomycin-susceptible MDR clinical E. coli strains except one (strain K71-77; *P* = 0.062) (Fig. 7A). In the bladder and kidneys, the counts (number of CFU per milliliter) were significantly reduced for 3/5 and 1/5 of the fosfomycin-susceptible strains, respectively (Fig. 7B and C). No significant difference in the counts (number of CFU per milliliter) between the treated and the vehicle groups was observed for the fosfomycin-resistant strain (P14-63) at all infection sites (Fig. 7A to C).

### Posttreatment studies.

No significant changes in the MIC of fosfomycin for colonies of selected isolates on day 2 and day 5 (*n* = 71) from any infection site of either the treatment or the vehicle group randomly picked from nonselective agar were observed. All strains retained their expected determinants of resistance to third-generation cephalosporins and/or carbapenems.

## DISCUSSION

### Pharmacokinetics/pharmacodynamics.

Our first objective was to perform PK/PD studies to find the predictive index for fosfomycin. In the mouse model, fosfomycin was rapidly absorbed after s.c. injection. After an almost negligible distribution phase, the drug was eliminated with a mean half-life of 28 min in plasma (Fig. 3). The elimination rate in mice was almost 10 times faster than that observed in humans, as is usually seen for drug kinetics in mice. No accumulation of the drug in serum is expected for the observed concentrations due to its short half-life in mice. Due to a significant variation in urinary drug concentrations and because it was not possible to measure the total mass of excreted drug, we did not use these data in the PK/PD analysis. Interestingly, the urine concentration curves of fosfomycin were similar for two different doses (30 mg and 7.5 mg), except at the time point of 15 min, where an approximately 4-fold difference in peak urine concentrations was observed (Fig. 3B). This could suggest saturable elimination in combination with first-order elimination. Other studies, in both humans and animal models, suggest that fosfomycin is eliminated in kidneys exclusively by glomerular filtration and is neither protein bound nor metabolized (29–35). However, there are some examples of similar findings in human studies, where a ceiling effect on excretion has been observed (36, 37). We believe that this phenomenon deserves further studies, especially with respect to the optimal dosing regimen in humans.

According to the results of time-kill studies, the bactericidal effect of fosfomycin was rapid (<2 h) and concentration dependent (Fig. 1 and 2). Regrowth after 24 h was also shown to be concentration dependent and was observed for all the concentrations below 16× to 32× MIC. Moreover, the MICs for the survivors increased 8- to 32-fold and were stable, indicating the development of resistance. This is in contrast to the lack of an increase in the MIC for randomly selected colonies posttreatment *in vivo*. Although no firm conclusions can be drawn with respect to the *in vivo* emergence of fosfomycin resistance in our experimental setup, the findings of the present study support previous findings indicating differences between the *in vitro* and *in vivo* emergence of fosfomycin resistance (38).

We used bloodstream drug concentrations in the PK/PD analysis. According to Frimodt-Møller (39), the serum PK/PD indices represent a more accurate predictor of drug levels and treatment activity in kidneys. For activity in bladder, a combination of urinary (lumen) and serum (bladder tissue) PK could be important. Our dosing regimens allowed variations in the magnitudes of the PK/PD indices. However, due to a high renal clearance in mice, the *T*_>MIC_ did not exceed 42% for the strain used in the study. We observed overall good activity for all dosing regimens, despite the relatively low percent *T*_>MIC_ for some doses (Fig. 4). Due to the differences in pharmacokinetics between mice and humans, we could not mimic all the parameters with one dose, especially when the fact that we were assessing both plasma and urine concentrations is taken into account. Mazzei et al. have reported that high fosfomycin urine concentrations (1,000 to 4,000 mg/liter) are achieved and remain at 100 mg/liter for at least 30 to 48 h (40), which is the pharmacokinetic basis for the oral 3-g single-dose regimen. In our dose fractionation study, we used a set of doses which appeared to be related to the standard human oral 3-g dose in different ways. In comparison with the PK of the standard human oral 3-g dose, a dose of 0.47 mg/mouse had a comparable plasma *C*_max_ of 22 mg/liter (41), a dose of 7.5 mg/mouse had a comparable plasma AUC of 212 h^−1^ (41), doses of 1.88 to 0.47 mg/mouse had comparable peaks in urine concentrations (40) (however, the concentrations declined faster), and doses of 15 to 30 mg/liter allowed the urine concentrations of fosfomycin to be retained at >100 mg/liter for the longest time compared to the other doses used in this study, while plasma *C*_max_ levels were comparable to those obtained with intravenous (i.v.) bolus doses in humans (42). The differences in PK parameters at different sites and for different doses can also be seen to be an advantage, as it would allow us to isolate the PK/PD parameters which are important for the successful treatment of UTIs with fosfomycin.

We used a proportion of the bactericidal effect approach, which allowed us to account for both noninfected/self-recovered kidneys and the good overall activity seen for all doses (Fig. 4). For urine and kidneys, the bactericidal effect was defined as the number of CFU per milliliter below the LOD, while for the bladder, the bactericidal effect was defined as the number of CFU per milliliter below the minimum count observed in the control group. Similar results for the number of CFU per milliliter in the bladder were previously observed in the same animal model with other antimicrobials (43). For all infection sites, the optimal PK/PD indices were AUC/MIC_0–72_ and *C*_max_/MIC.

Published data regarding the appropriate PK/PD index for fosfomycin are somewhat inconsistent. Some authors (23, 24) consider *T*_>MIC_ to be an appropriate index; however we, along with others (22, 26), found the AUC/MIC and/or *C*_max_/MIC to be more appropriate. This may be because for most of the strains, the dose allowed the fosfomycin concentrations to remain above the MIC for a substantial amount of time. Treatment failures may happen due to the emergence of an inherently resistant subpopulation. VanScoy et al. (25) showed *in vitro* that the time above the inherent resistance inhibitory concentration (32× to 64× MIC, in their case) appeared to be the optimal PK/PD index; our results do not contradict this hypothesis.

### *In vivo* activity studies.

In order to balance comparable serum concentrations and prolonged fosfomycin concentrations in urine, a dose of 15 mg/mouse administered twice (q36h) was considered to be the most effective and, in terms of surface area and as discussed above, comparable to the standard human dose of 3 g fosfomycin used for the treatment of UTIs. This dose is also expected to sustain fosfomycin concentrations in urine of >100 mg/liter for the longest possible time (≈10 h) and was further used for the treatment studies. The decrease in bactericidal effect was relative to the MICs for the strains. For the isogenic strain NU14 derivative (DA6328) with an MIC of 128 mg/liter, the dose resulted in a reduction in the number of CFU per milliliter in urine for some mice (3/6), which was, overall, statistically nonsignificant. For the same strain, no reduction in the number of CFU per milliliter in the kidneys and bladder was observed (Fig. 6).

The second objective of the study was to evaluate the activity of fosfomycin against MDR E. coli *in vivo*. Fosfomycin significantly reduced the number of CFU per milliliter in the urine and bladder for most of the isolates. Although not all the isolates showed statistically significant reductions in the number of CFU per milliliter in the bladder and kidneys, nonsignificant tendencies toward reductions could still be observed (Fig. 7). For cases with low statistical significance, the results could be explained by the lower total number of CFU per milliliter per organ compared to that in urine, and thus, the difference in the reduction (between the treatment and vehicle-treated groups) was smaller. The high proportion of low counts (number of CFU per milliliter) of some strains in the kidneys in the vehicle-treated group (which could be interpreted as indicating either that the animals were not infected or self-recovered) is considered a limitation of the model. We believe that this situation causes a lower level of statistical significance when the two-tailed Mann-Whitney test for the counts (number of CFU per milliliter) in kidneys is used. These results are in concordance with those of other studies (43, 44) implementing the same animal model but using different antimicrobials and, thus, can be considered to be due to the limitations of the model and not the action of fosfomycin itself. The results indicate that fosfomycin might have a potential for use in the treatment of upper UTIs (for strains with lower MICs and likely by use of a dose or route of administration different from the standard 3 g oral single dose used in the clinical setting), but this requires further studies.

The colony counts of the resistant clinical isolate harboring *fosA* (P14-63) were not significantly reduced *in vivo* in this study, despite the high plasma and urinary concentrations (Fig. 7). In accordance with this finding, the time-kill studies also showed that the strain was not inhibited even by concentrations exceeding 32× MIC (>16,384 mg/liter). The same time-kill pattern for isolates with plasmid-mediated fosfomycin resistance has also been previously observed (45). Examples of colinked *fosA* and ESBL determinants have already been reported (46, 47). However, *ad interim*, the global rates of susceptibility to fosfomycin remain high, including for ESBL- and carbapenemase-producing Enterobacteriaceae (48, 49).

In conclusion, our observations support the notion that fosfomycin is a promising option for the treatment of uncomplicated UTIs caused by MDR E. coli. The proportions of susceptible isolates among the subgroup of ESBL or carbapenemase producers remain high globally (19, 48, 49). The optimal PK/PD indices included AUC/MIC and *C*_max_/MIC. The dose of 15 mg/mouse twice (q36h) demonstrated a good effect against clinical isolates. To our knowledge, this is the first *in vivo* study reporting the successful treatment of UTIs caused by carbapenemase-producing E. coli with fosfomycin.

## MATERIALS AND METHODS

### Bacterial strains and chemicals.

The fosfomycin-susceptible clinical uropathogenic E. coli strain (UPEC) NU14 (38) was used to evaluate the effect of different doses of fosfomycin for the PK/PD study. The strain has been used in a number of studies of UPEC (50) as well as in the UTI model (51).

Three isogenic E. coli strains with decreasing fosfomycin susceptibility, DA6313, DA6328, and DA6401, derived from NU14 (38), were included for evaluation of the selected doses in the UTI model. These strains have increased MICs against fosfomycin due to chromosomal mutations/deletions (38), including a deletion in *ptsI* (DA6313), a *glpT* missense mutation (DA6328), and a missense mutation in *uhpT* (DA6401). Further, one fosfomycin-resistant and five fosfomycin-susceptible clinical ESBL-, plasmid-mediated AmpC-, and/or carbapenemase-producing E. coli isolates were used to further evaluate the efficacy of the selected doses. All isolates used in the study are listed in Table 1.

The MIC of fosfomycin was determined by agar dilution using Mueller-Hinton (MH) agar (Oxoid, Waltham, MA) with the addition of 25 mg/liter glucose-6-phosphate (G6P; Sigma-Aldrich, St. Lois, MO), as recommended by EUCAST (52) and by CLSI guidelines (53). Fosfomycin powder (fosfomycin disodium; batch no. 20120323) was supplied by Ningbo Honor Chemtech Co., Ltd., Ningbo, China. Fosfomycin disodium solution has been reported to be stable for both a short period (24 h) and longer periods of up to 14 days (54, 55) and to have a long shelf-life (2.9 × 10^7^ h) when stored in dry powder form (56). Moreover, the potency of the powder was regularly reevaluated during the study by agar dilution MIC testing of both a fresh solution and a solution that had been stored overnight at 4°C, and it remained stable. A stock solution was prepared from dry powder prior to each experiment. E. coli ATCC 25922 (American Type Culture Collection, Manassas, VA) was used as a quality control organism in the susceptibility testing and for the bioassay evaluating fosfomycin concentrations in the PK studies.

For the isolates used in the treatment study, the multilocus sequence type and resistance determinants were explored by whole-genome sequencing (Illumina, San Diego, CA) and analysis of the sequence at the Centre for Genomic Epidemiology (https://cge.cbs.dtu.dk/services/CGEpipeline-1.1).

Type 1 fimbria production, essential for establishment of a successful murine UTI (44), was confirmed for all clinical MDR strains using a mannose-sensitive agglutination of yeast cells (Saccharomyces cerevisiae, lot BAD0641-2; Idun Industri, Norway) as described before (57, 58).

### *In vitro* time-kill studies.

Time-kill studies were performed for all isolates as described previously (59), with one minor modification. The bacterial suspension was added to tubes with fosfomycin instead of addition of fosfomycin to the bacterial suspension. This reverse order was introduced to avoid possible problems with fosfomycin solubility in the concentrated stock solution. The modification did not result in a change of the final bacterial density or the fosfomycin concentration in the test tube. Comparison of the time-kill curves obtained with the original protocol and the modified protocol did not show any difference with the fosfomycin-sensitive isolates (data not shown). Briefly, colonies from an overnight culture were suspended in 0.9% saline to an optical density at 546 nm of 0.13. One milliliter of the bacterial suspension was added to a tube containing 9 ml MH broth (Mueller-Hinton II broth; catalog number BBL 212322; Becton Dickinson, Franklin Lakes, NJ) with 25 μg/ml G6P (Sigma-Aldrich), resulting in a bacterial density of 1 × 10^7^ CFU/ml. The bacterial suspension was incubated at 37°C with shaking (140 rpm) for 25 min, and 1 ml was added to tubes containing 19 ml of fosfomycin at different concentrations proportional to the MIC for each strain in MH broth with 25 mg/liter G6P (Sigma-Aldrich), resulting in a bacterial density of approximately 5 × 10^5^ CFU/ml. Viable counts were determined at time points of 0, 0.5, 1, 2, 4, and 24 h after the start of antimicrobial exposure using spot serial dilution (60).

Single NU14 colonies appearing on the MH agar plates used for determination of the number of CFU (for each fosfomycin concentration, including a negative control, at time points of 0 h, 4 h, and 24 h) were resuspended and tested for a change in the fosfomycin MIC by agar dilution, as described above. The isolated subpopulations were further passaged five times on MH agar medium (Oxoid, Waltham, MA) to evaluate the stability of the fosfomycin MIC.

### PK studies.

Three studies of the pharmacokinetics (PK) of fosfomycin in the bloodstream and urine were performed in outbred female albino OF-1 mice (weight, ∼30 g; Charles River Laboratories, Chatillon-sur-Chalaronne, France) given a single subcutaneous (s.c.) dose of 0.75, 7.5, and 30 mg fosfomycin per mouse, respectively. Blood was sampled by periorbital cut-down, and urine was collected directly in an Eppendorf tube by placing the tube over the orifice and gently tapping the mouse on the abdomen. Samples were drawn at 15, 30, 60, 120, 180, and 240 min after dosing. Three mice were sampled at each time point. Blood was sampled in EDTA-coated Eppendorf tubes (Eppendorf, Hamburg, Germany), the tubes were centrifuged at 1,800 × *g*, and plasma was transferred to fresh Eppendorf tubes and stored at −80°C. Urine was also sampled in Eppendorf tubes and stored at −80°C. Fosfomycin concentrations were measured by a bioassay using the fosfomycin-susceptible E. coli ATCC 25922 strain. A bacterial suspension (10^6^ CFU/ml) was floated on MH agar plates (Oxoid), and paper discs (Oxoid) were placed on the inoculated agar. Twenty microliters of fosfomycin standards (1.1, 3.3, 11, 33, and 100 mg/liter) spiked in pooled mouse plasma or urine from untreated mice was pipetted onto each disc. The same procedures were performed with triplicate samples from plasma and urine from treated mice. After overnight incubation, the inhibition zone diameters were measured and the concentrations were calculated from standard curves using regression analysis. For concentrations higher than 100 mg/liter, samples were diluted in plasma or urine until measured values below the maximum standard were obtained. The standard concentrations showed a day-to-day variation of <10%.

### Calculation of dosing regimens.

Doses for the *in vivo* activity study were designed to vary the *T*_>MIC_ and AUC/MIC_0–72_. Through interpolation and extrapolation of the PK data, the exponential equation describing the concentration curve was estimated. Dose-dependent PK indices (AUC/MIC_0–72_, *T*_>MIC_, *C*_max_/MIC) were computed on the basis of the total drug concentrations. *T*_>MIC_ (percent per 72 h) was calculated as the percentage of time that the drug concentration was above the MIC for the test strain (NU14; MIC, 1 mg/liter) during the treatment period (72 h); indices related to concentration dependence (*C*_max_/MIC) were calculated on the basis of the highest concentrations observed experimentally 15 to 240 min after the s.c. dose (for the doses of 30 mg and 7.5 mg) or through interpolation and extrapolation (for the other doses). Since the maximal drug concentrations in plasma were registered at the first measurement time point (15 min after injection), leading to the assumption that the real peak in the fosfomycin concentration might have occurred before the first measurement, we additionally used the “*T*_>MIC_ (in hours) after the first dose” as a surrogate marker for *C*_max_/MIC after the single dose as an index by assuming that the longer the *T*_>MIC_ (in hours) after the first dose is, the higher the *C*_max_/MIC is. As the total number of doses administered within 72 h varied significantly (1 to 12 doses per 72 h; Table 2), the *T*_>MIC_ (in hours) after the first dose did not show a linear relationship with the *T*_>MIC_ (percent per 72 h).

Indices considering both time and concentration (AUC/MIC_0–72_) were calculated as the size of the area under the concentration-time curve divided by the MIC using the trapezoidal rule (regular). All calculations were performed in GraphPad Prism (version 7) software (GraphPad Software, San Diego, CA). The relevant PK indices for the applied dosages are listed in Table 2.

### PK/PD, virulence, and *in vivo* activity studies.

The virulence of the strains was confirmed *in vivo* in the murine UTI model before proceeding to the treatment studies, as previously described (43, 44). Briefly, immunocompetent outbred albino female mice (OF-1; Charles Rivers Laboratories, Chatillon-sur-Chalaronne, France) were used. Three days prior to inoculation, the drinking water was replaced with 5% glucose solution (Sigma-Aldrich). On the inoculation day, mice were given ibuprofen (Nurofen Junior; Novartis, Basel, Switzerland) orally and tiletamine-zolazepam (Zoletil; Virbac SA, Carros, France) plus butorphanol tartrate (Torbugesic; Fort Dodge Laboratories, IA, USA) subcutaneously. The bladders of anesthetized mice were inoculated with 50 μl a bacterial suspension containing approximately 10^9^ CFU/ml. Transurethral inoculation was performed with a sterilized plastic catheter (Becton Dickinson, NC, USA), which was further retracted. Urine was collected from day 2 to control for the establishment of infection. Mice were observed for any signs of pain or illness during the next 3 days. On day 5, urine was collected from the mice by gently pressing on the abdomen. The mice were then euthanized by cervical dislocation and the remaining urine was added to the tubes. Subsequently, the emptied bladder and both kidneys were aseptically removed. The urine samples were processed on the same day by spotting (20 ml) of a series of 10-fold dilutions in duplicate (spot dilution technique) on bromothymol blue agar plates (Statens Serum Institut, Copenhagen, Denmark). The organs were homogenized in a Tissue Lyser apparatus (Qiagen, Ballerup, Denmark); organ homogenizing was performed by adding 0.9% saline to the organs until the total volume of 500 µl for bladders and 100 µl for two kidneys was reached. Tissue homogenates were stored frozen and used to determine viable bacterial counts on the next day as described above for urine. Colony counts on plates were performed after 18 to 24 h of incubation at 37°C in ambient atmosphere.

The NU14 strain was used for performing the PK/PD study, where the effect of the calculated fractioned doses was evaluated. Infection in the murine model was initiated as described above. Six to 18 mice per group were used for investigation of each dose (43, 44). At 24 h postinoculation (day 2), after the collection of urine, mice were treated subcutaneously with fosfomycin (total doses of 2.82 to 30 mg per mouse for a treatment period of 72 h at a dosing frequency ranging from a single dose to dosing q6h; Table 2) or saline on days 2 to 4. On day 5, the colony counts in the urine, bladders, and kidneys were determined as described above.

To evaluate the *in vivo* activity of a selected dose on the basis of the results of the PK/PD study, the effect of treatment against three isogenic strains of NU14 and clinical MDR E. coli strains with different fosfomycin MICs was tested (Table 1). The *in vivo* activity studies were performed as described above. For each strain, animal groups were treated with either 15 mg fosfomycin q36h per mouse (7 mice) or vehicle (6 or 7 mice).

For the clinical isolates, bacterial populations that survived during the *in vivo* fosfomycin treatment were collected. Two colonies from both the treatment and vehicle groups and, when possible, from the same mouse were collected from plates on which urine was seeded on day 2 and from plates for the colony counts for every site of infection (urine, bladder, and kidneys) on day 5. Further, the fosfomycin MICs for these colonies were compared to the MICs for strains isolated from the same mouse on day 2. PCR was used to confirm the presence of ESBL, plasmid-mediated AmpC, and carbapenemase genes as described before (61–63). The fosfomycin MIC for the surviving strains was determined by agar dilution, as described above.

### Statistical analysis.

GraphPad Prism (version 7) software (GraphPad Software, San Diego, CA, USA) was used for the PK/PD analysis. The relationship between the effect and PK/PD indices was analyzed according to a sigmoidal Hill function (four-parameter dose-response curve). For each PK/PD index, the data were fitted simultaneously for all distinct doses using nonlinear regression with the ordinary least-squares (OLS) algorithm. Due to the high proportion of mice in all dose groups with reductions in the number of CFU per milliliter below the LOD, we used the cumulative effect, measured in percent (defined as the bactericidal effect), as the PD endpoint (64). This approach accounted for the high proportion of cases with colony counts below the LOD and the 40% proportion of nonaffected/self-recovered kidneys in the control group. For urine and kidneys, the bactericidal effect was defined as the counts (number of CFU per milliliter) below the LOD, while for bladder counts (number of CFU per milliliter), the bactericidal effect was defined as the proportion of counts (number of CFU per milliliter) that were lower than that for the control group.

In the *in vivo* activity study with clinical strains, the median counts (number of CFU per milliliter) were compared pairwise (between the treatment and vehicle-treated groups, separately for each strain) using the Mann-Whitney test (two-tailed) with a significance level of a *P* value of ≤0.05 (GraphPad Prism [version 7] software). For each strain, a separate control group treated with vehicle was used. We chose not to perform the correction for multiple comparisons (65).

### Ethical considerations.

The animal experiments were carried out at the animal facility at the Statens Serum Institute, Copenhagen, Denmark, and approved by the Danish Animal Experimentation Inspectorate (no. 2014-15-0201-00204).
